# BioLadder: A bioinformatic platform primarily focused on proteomic data analysis

**DOI:** 10.1002/imt2.215

**Published:** 2024-06-20

**Authors:** Yupeng Zhang, Chunyuan Yang, Jinhao Wang, Lixin Wang, Yan Zhao, Longqing Sun, Wei Sun, Yunping Zhu, Jingli Li, Songfeng Wu

**Affiliations:** ^1^ Beijing Qinglian Biotech Co., Ltd. Beijing China; ^2^ State Key Laboratory of Proteomics Beijing Proteome Research Center, National Center for Protein Sciences (Beijing), Beijing Institute of Lifeomics Beijing China

## Abstract

BioLadder (https://www.bioladder.cn/) is an online data analysis platform designed for proteomics research, which includes three classes of experimental data analysis modules and four classes of common data analysis modules. It allows for a variety of proteomics analyses to be conducted easily and efficiently. Additionally, most modules can also be utilized for the analysis of other omics data. To facilitate user experience, we have carefully designed four different kinds of functions for customers to quickly and accurately utilize the relevant analysis modules.

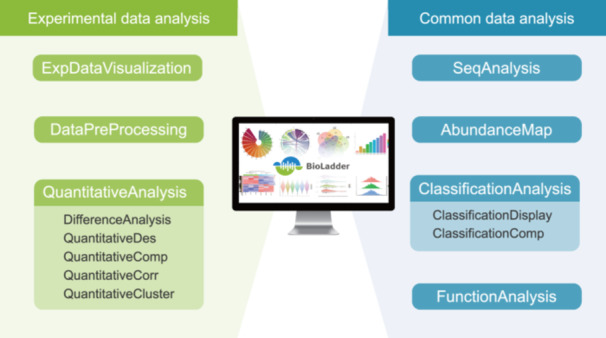

To the Editor,

In recent years, the vigorous development of multiomics research has generated massive amounts of data, and in‐depth data analysis and mining have become an important feature of life science research [1, 2]. Bioinformatics has become one of the most commonly used research tools, playing a pivotal role in life science research.

However, bioinformatics research requires programming training, which may not be a strong suit for those researchers that focus on scientific questions. Moreover, even some researchers have coding skills, who still need to invest considerable time and effort in coding to complete the analysis, which undoubtedly leads to delays in related work.

Online analytical platforms are undoubtedly the first choice for researchers, as they do not require additional installation and preparation work. Simply opening a web page and uploading data for analysis can greatly accelerate the pace of life science research. Currently, there are many similar online data analysis platforms, including some specialized omics data analysis platforms, such as ImageGP [3], Sangerbox [4], Majorbio Cloud [5], OmicStudio [6], OmicsSuite [7], OmicsAnalyst [8], and so forth. However, most of these analytical platforms were developed based on the needs of genomics and transcriptomics, and almost none are specifically designed for proteomics.

The proteome is translated from the transcriptome and not only possesses the expressive properties of the transcriptome, but also includes additional properties, such as modifications and interactions [9, 10]. In terms of qualitative and quantitative experimental techniques, proteomics is far more complex than genomics and transcriptomics, which imposes additional requirements on data analysis. In recent years, with the advancement of technology, proteomics has gradually played an increasingly important role in medical research [11, 12], leading to a growing and diverse demand for protein data analysis.

Here, we provide the BioLadder bioinformatics platform (https://www.bioladder.cn/), which not only offers some conventional analytical tools but also provides commonly used proteomics analysis tools, including experimental result visualization, sequence‐level analysis, expression data analysis, and functional analysis. Some of the tools are newly developed and currently have no equivalent tools available online.

## MODULE DESIGN FOR PROTEOMIC DATA ANALYSIS

**Figure 1:**
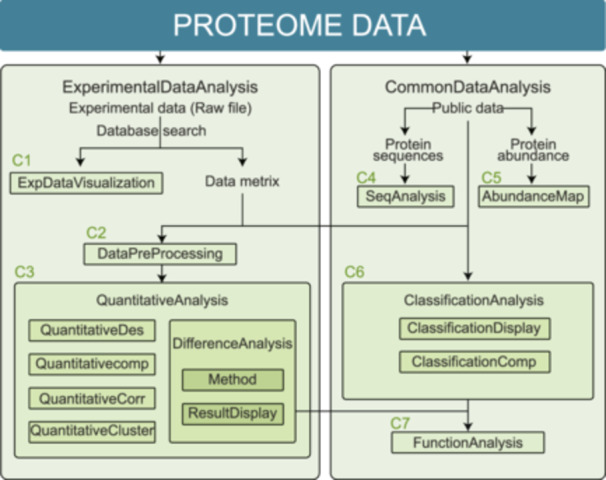
BioLadder module classes in the proteome data analysis framework. BioLadder consists of over 50 analysis modules, which belong to two main categories (ExperimentalDataAnalysis and CommonDataAnalysis) and seven classes (C1–C7 in the figure).

Proteomic data analysis can be divided into two main categories (Figure 1): (1) Experimental data analysis: Analysis related to proteomics experimental data, including the analysis of experimental data, expression matrix data analysis, and so forth (Classes 1–3); (2) Common data analysis: Analysis not dependent on proteomics experimental data, including protein sequence analysis, as well as some general classification and functional analysis, and so forth (Classes 4–7).

The seven classes are outlined as follows:

### Class 1. ExpDataVisualization

Experimental data visualization currently includes two modules (CoverageBar and Pep2ProMap), which display the coverage of proteomic identification peptides to proteins, as well as the information on protein digestion sites.

### Class 2. DataPreProcessing

Data preprocessing includes data format conversion, normalization, imputation, and so on. It is an important part for the following analysis.

### Class 3. QuantitativeAnalysis

Quantitative comparison entails analyzing the quantitative results of each protein and is the most prevalent type of analysis module, subdivided into five groups: (1) DifferenceAnalysis: Differential analysis encompasses differential calculation, FDR (False Discovery Rate) correction, and the visualization of differential results, such as volcano plots, ROC (Receiver Operating Characteristic) curves, and so forth. These modules are capable of both differential calculation and result presentation; (2) QuantitativeDes: Quantitative data description includes creating scatter plots, density plots, distribution bar or line graphs, as well as coefficient of variation (CV). These modules are designed to describe the distribution, density, and other features of quantitative data; (3) QuantitativeComp: Quantitative data comparison includes bar graphs, heat maps, box plots, and so on. These modules are primarily utilized to compare the quantitative differences or variations among different samples or genes; (4) QuantitativeCorr: Quantitative data correlation includes correlation heat maps, correlation matrix graphs, and more. These modules calculate the quantitative correlation between samples or genes to reveal the relationships among samples or genes; (5) QuantitativeCluster: Quantitative clustering includes dimensionality reduction methods, like, PCA (Principal Component Analysis), T‐SNE (T‐Distributed Stochastic Neighbor Embedding), UMAP (UniformManifold Approximation and Projection) for dimension reduction, trend analysis of multiple data sets, TreeDiagram, and so on. These modules generally utilize algorithms for dimensionality reduction or other distance calculation methods to cluster and analyze samples or genes.

### Class 4. SeqAnalysis

Sequence analysis refers to analyses that can be completed based on protein sequences, including multiple sequence alignment, sequence motif analysis, calculation of protein physicochemical properties, and so forth.

### Class 5. AbundanceMap

The abundance chart offers a convenient way to query and display reference quantitative data for body fluids (currently including blood and urine).

### Class 6. ClassificationAnalysis

Classification analysis consists of two groups: classification display and classification comparison: (1) Classification display involves presenting the differences in results of different types after classification using scatter plots, pie charts, area charts, and so forth; (2) Classification comparison entails comparing results of different types using VennChart, Sankey diagrams, Radar charts, and other visualizations.

### Class 7. FunctionAnalysis

Function analysis focuses on visualizing enrichment results based on Gene Ontology, as well as drawing interaction network diagrams.

Therefore, the analysis modules included in Bioladder cover experimental data analysis in proteomics research, as well as multiple modules for public sequence data analysis (Table S1). These analysis modules can meet most of the data analysis needs of researchers in the field of proteomics.

## SPECIFIC PROTEOME DATA ANALYSIS MODULES

In response to the needs of proteomics research, we have developed several proteome data visualization modules (Table S2), such as (1) coverage analysis of peptide segments in protein sequences, including the CoverageBar and Pep2ProMap modules. These modules are primarily designed for presenting Lip‐MS (Limited Proteolysis‐Mass Spectrometry) experimental results, but can also be used to display identification data from any proteomics experiment; (2) analysis and visualization of quantitative data distribution, including the CV curve and SumCurve modules. Users can utilize these modules to examine the variability and abundance curves of quantitative data; (3) quantification data and marked proteins, including the AbundancePoint and BodyFluidMap modules. The former allows users to input their own quantitative data and specify proteins, while the latter enables users to query the quantitative information of specific proteins in the body fluid database (currently including blood and urine).

We believe that these proteome data visualization modules will meet the demands of proteomics research and provide valuable insights for researchers.

## CONVENIENT AND USER‐FRIENDLY DESIGN

**Figure 2:**
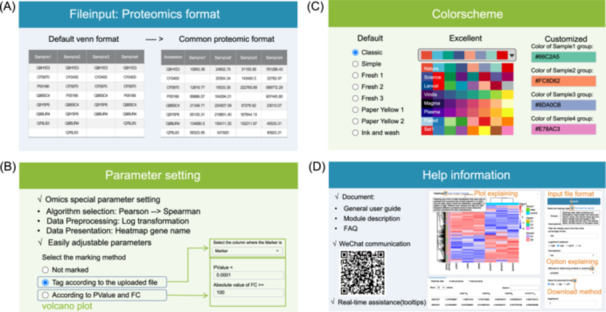
Four convenient and user‐friendly designs in BioLadder. (A) An example of a popular proteomic file format as the default input format (Venn plot). (B) Specialized default parameters for proteomics, including algorithm selection, data preprocessing, and presentation. Diverse and extensive adjustment methods (volcano plot as an example). (C) Three different groups of color schemes. (D) Comprehensive help information (three kinds of documents and WeChat communication), and convenient real‐time assistance (heatmap plot as an example).

To enable users in omics research to utilize our online analysis platform in the most convenient and efficient manner, we have meticulously designed various aspects, including input file formats (Figure 2A), parameter settings (Figure 2B), color schemes (Figure 2C), and so on. We provide help documentation, WeChat customer service, and real‐time tooltips to make it easy for customers to access relevant help information (Figure 2D). Only part of these designs can be implemented in current online cloud platforms (Table S3).

## SIMPLIFIED INPUT FORMAT

Many data analysis methods are universal across different fields with its own input data format, which may not be commonly used in the field of proteomics. Proteomics data may require some transformation to facilitate the corresponding analysis. Therefore, in our design, we provide conversion modules for different types of data (e.g., converting between long and wide formats) and design some modules to directly support common proteomics formats. For example, in the Venn diagram module, users can not only input commonly used Venn format data but also directly input quantitative matrix data tables (i.e., usually used in proteomics) for analysis. Additionally, it could also filter out some data below a certain minimum quantitation value, which helps eliminate results that may be caused by noise.

## SPECIALIZED DEFAULT PARAMETERS FOR PROTEOMICS, DIVERSE, AND EXTENSIVE ADJUSTMENT METHODS

To meet the specific requirements of proteomics data analysis, we have established suitable default parameters for some modules to minimize the need for parameter adjustments as much as possible.

First, in terms of algorithms, we have adjusted default parameters based on the characteristics of proteomics data. For instance, in correlation calculations, due to the nature of expression data, a few highly abundant proteins may significantly impact the default Pearson correlation calculation. Therefore, in those modules that involved correlation calculations, we have defaulted to using Spearman rank correlation for computation, which were adopted in many proteomics‐related studies as well [13, 14, 15]. Furthermore, considering that there is often significant variation in the identified protein numbers of different samples, conventional normalization methods may inevitably introduce bias. To address this issue, we have incorporated a method called median normalization of common proteins in the normalization module.

Second, in data preprocessing, we made some adjustments based on the data characteristics of proteomics. For example, as most genes tend to be relatively low abundance, directly plotting quantitative distributions often results in most proteins being concentrated in low abundance, which makes the differences between samples hard to discern [13, 14, 15]. Hence, in modules such as box plots, violin plots, and kernel density plots, we have directly set the default to require logarithmic transformation, allowing for clear visualization of quantitative data variances across different samples without any parameter modifications.

Furthermore, we also made some special default parameters in data presentation. For instance, in heatmap analysis, with genes typically numerous on the y‐axis, displaying gene names can often be illegible. Therefore, we have defaulted to display only sample names and omitting gene names for better clarity.

In addition, to cater to user preferences, we have incorporated easily adjustable parameters in several modules, empowering users to customize their display results. For example, in volcano plot analysis, we have included two types of point annotation methods: (1) Customizing protein markers based on a designated marker column in the uploaded file; (2) Batch marking based on *p* value and fold change thresholds. Similarly, in box plot analysis, users can choose whether to add hypothesis test labels between different groups. We have also devised custom options allowing users to selectively add hypothesis test labels to specific group comparisons (e.g., only annotating significant results or comparisons of particular interest).

## POWERFUL COLOR SCHEME

Color scheme is a crucial aspect of data visualization, as improper color combinations can significantly reduce the effectiveness of visualizations.

To address this problem, we have configured default color schemes in all modules, including some default color schemes from R packages or ggplot2 (https://github.com/tidyverse/ggplot2), ensuring users can immediately create refined graphics without additional steps.

Furthermore, more than half the modules have incorporated additional color schemes sourced from commonly used excellent color schemes in literature or journals, such as Nature, Science, and Lancet (ggsci: https://github.com/nanxstats/ggsci).

For users with specific requirements, we offer the option to customize colors. Users can select colors directly using color palettes or precisely modify color configurations by adjusting color codes, enabling them to customize colors for each sample or group based on their preferences and esthetics.

These three functionalities provide our modules with powerful color customization capabilities, catering to various user needs and allowing users to quickly complete color customization according to their preferences.

Additionally, certain modules with unique characteristics utilize special color schemes. For example, the volcano plot module typically only requires three colors for upregulation, downregulation, and nonsignificance, so a color picker is used to set up the tricolor scheme.

## COMPREHENSIVE HELP INFORMATION, CONVENIENT REAL‐TIME ASSISTANCE

To ensure users can smoothly utilize our modules for data analysis, we provide helpful information from multiple perspectives in the “User Guide.” First, we offer an introduction to provide an overview of the website structure and functionalities. Second, we have a “Frequently Asked Questions” page that compiles the most common inquiries. Third, detailed documentation is provided for each module. Additionally, we offer a WeChat communication group where users can directly consult our staff about encountered issues.

Furthermore, besides commonly used parameter settings, we have added tooltips for instant assistance, allowing users to access helpful information on parameter settings at any time to help accurately configure the corresponding parameters. For instance, in the heatmap module, four types of tooltips are provided: (1) Tooltip for input file details, including file content explanation, maximum file limits, and file formats; (2) Tooltip for dropdown selection boxes, explaining the meaning of each option; (3) Tooltip for download formats, providing download instructions and graphical explanations of download settings; (4) In the top left corner of result plots in most modules, a “Text Tutorial” link is provided, along with a tooltip explaining the plot, allowing customers to quickly understand the plot's significance. These tooltips enable users to easily access helpful information and seamlessly continue with configuration and data analysis.

## METHODS

BioLadder's user interface is engineered upon the Vue.js framework, offering a robust and interactive client‐side experience. The server‐side architecture is meticulously crafted utilizing the Laravel framework, known for its expressive syntax and robust features. The platform's data persistence is managed by MySQL, ensuring reliable and efficient data management. The analytical functionalities are delivered through a synergistic integration of JavaScript for dynamic web interactions, Shiny for creating interactive web applications, and a selection of R packages optimized for statistical analysis and graphics, enabling sophisticated data processing and visualization within the proteomics domain.

## AUTHOR CONTRIBUTIONS

Songfeng Wu, Jingli Li, and Yunping Zhu conceived the idea of developing the BioLadder platform. Yupeng Zhang completed the construction of the platform and the implementation of various modules. Chunyuan Yang built and maintained computing services and wrote manuscript. Jinhao Wang assisted in completing some of the development work, while Lixin Wang assisted in conducting research and promotion. Yan Zhao, Longqing Sun, and Wei Sun assisted in the testing and proposed modification suggestions. All authors have read the final manuscript and approved it for publication.

## CONFLICTS OF INTEREST STATEMENT

Yupeng Zhang, Jinhao Wang, Lixin Wang, Yan Zhao, Longqing Sun, Wei Sun, Jingli Li, and Songfeng Wu are employees and researchers of Qinglian Biotech Co., Ltd. The remaining authors declare no conflict of interest.

## Supporting information


**Table S1**: BioLadder modules in the proteome data analysis framework.
**Table S2**: The possible application for each new developed modules.
**Table S3**: Comparison of BioLadder convenient and user‐friendly designs in different cloud platforms.

## Data Availability

The data and scripts used are saved in GitHub https://github.com/sz-zyp/BioLadder2024. Supporting Information (tables, scripts, graphical abstracts, slides, videos, Chinese translated version, and update materials) is available online DOI or http://www.imeta.science/. Data sharing is not applicable to this article as no new data were created or analyzed in this study.
